# Association of Placental Growth Factor with the risk of adverse pregnancy outcomes: a prospective cohort study in Chinese pregnant women

**DOI:** 10.3389/fendo.2025.1674540

**Published:** 2025-10-02

**Authors:** Tai-Shun Li, Yuan Wang, Ya Wang, Hui-Rong Tang, Hong-Lei Duan, Guang-Feng Zhao, Jie Li, Ya-Li Hu

**Affiliations:** ^1^ Department of Obstetrics and Gynecology, Nanjing Drum Tower Hospital, The Affiliated Hospital of Nanjing University Medical School, Nanjing, China; ^2^ Medical Statistics and Analysis Center, Nanjing Drum Tower Hospital, The Affiliated Hospital of Nanjing University Medical School, Nanjing, China

**Keywords:** preeclampsia, small for gestational age, placental growth factor, cohort, adverse pregnancy outcomes

## Abstract

**Background:**

Adverse pregnancy outcomes, such as preterm birth, preeclampsia (PE), small for gestational age (SGA), pose significant risks to maternal and neonatal health and contribute to healthcare burdens. Placental Growth Factor (PIGF), a key pro-angiogenic biomarker involved in placental development, has been implicated in the pathophysiology of these complications. This study aimed to investigate the association between maternal serum PIGF levels and adverse pregnancy outcomes in a prospective cohort.

**Methods:**

We conducted a cohort study involving 5,870 women with singleton pregnancies enrolled at Nanjing Drum Tower Hospital from January 2017 to September 2020. Participants were followed from early pregnancy (≤14 gestational weeks) through delivery. Logistic regression models were used to evaluate the associations between serum PIGF levels (measured at 11–14 gestational weeks) and adverse pregnancy outcomes, reported as adjusted odds ratios (ORs) with 95% confidence intervals (CIs). Dose–response relationships were assessed using restricted cubic spline analysis.

**Results:**

Serum PIGF concentrations in early pregnancy were inversely associated with PE (OR = 0.97, 95% CI:0.96 – 0.98), preterm PE (OR = 0.96, 0.94 – 0.98), SGA <10th percentile (OR = 0.99, 0.98 – 0.99) and SGA <3rd percentile (OR = 0.98, 0.97 – 0.99). Expressed as multiples of the median (MoM), PIGF showed stronger associations with these outcomes, including PE (OR = 0.32, 0.21 – 0.48), preterm PE (OR = 0.23, 0.09 – 0.56), SGA <10th percentile (OR = 0.67, 0.54 – 0.83) and SGA <3rd percentile (OR = 0.43, 0.29 – 0.64), compared with its absolute concentrations. Notably, PIGF demonstrated a consistent inverse association with PE across different modes of conception, including spontaneous pregnancies (OR = 0.97, 0.96 – 0.98) and those conceived via ovulation induction or *in vitro* fertilization (OR = 0.95, 0.92 – 0.97). The highest predictive performance for PE was observed between 28–34 gestational weeks, with an area under the curve (AUC) of 0.79 (95% CI: 0.77 – 0.81). Additionally, dose–response analysis revealed nonlinear associations between PIGF levels and risks of SGA <10th and SGA <3rd.

**Conclusion:**

This cohort study reinforces the inverse association between maternal PIGF levels and the risks of PE and SGA. The findings highlight the potential clinical utility of PIGF as a gestational age–specific biomarker in prenatal risk stratification.

## 1 Introduction

Adverse pregnancy outcomes such as preeclampsia (PE), preterm birth, and small for gestational age (SGA) pose significant threats to maternal and neonatal health, and contribute to increased perinatal morbidity and mortality worldwide (1–4). Numerous maternal risk factors have been implicated, including advanced maternal age, elevated pre-pregnancy body mass index, chronic hypertension, renal dysfunction, and autoimmune diseases (5–8). However, identifying early biomarkers to predict and manage these complications remains a major clinical priority.

Placental Growth Factor (PIGF), a pro-angiogenic protein secreted by the placenta, plays a central role in placental vascular development and has garnered attention as a potential biomarker for pregnancy complications (9). Several studies have shown that low maternal serum PIGF levels are associated with an increased risk of PE (10–12), low birth weight, and fetal growth restriction (FGR) (13). In contrast, elevated PIGF concentrations have been linked to a reduced risk of spontaneous preterm birth (14). Nevertheless, the predictive performance of PIGF remains variable across studies, potentially due to differences in population characteristics, gestational timing of sampling, and methodological heterogeneity (15–17).

Importantly, limited evidence exists regarding the performance of PIGF across different modes of conception—such as spontaneous pregnancy, ovulation induction (OI), and *in vitro* fertilization (IVF)—and whether its predictive utility varies according to gestational age at measurement. Moreover, few studies have systematically characterized the dose–response relationships between PIGF and pregnancy outcomes, or assessed gestational age–specific predictive performance using standardized multiples of the median (MoM) values.

To address these gaps, we utilized data from our large prospective cohort study to investigate the association between maternal serum PIGF levels and a spectrum of adverse pregnancy outcomes. We examined dose–response patterns, assessed predictive performance across gestational age windows, and performed subgroup analyses stratified by mode of conceptions. Our findings aim to generate evidence that may inform future applications of PIGF in individualized risk assessment and screening strategies during early and mid-pregnancy.

## 2 Materials and methods

### 2.1 Study design and participants

This prospective, longitudinal cohort study included 5,870 singleton pregnant women who were admitted to Nanjing Drum Tower Hospital between January 2017 and September 2020. Participants were followed from early pregnancy (within 14 gestational weeks (GW), defined by a crown–rump length of 45–84 mm) through delivery. The study aimed to identify predictive factors for PE and collected comprehensive data on baseline characteristics, biochemical and biophysical markers, as well as maternal and fetal outcomes. Ethical approval was obtained from the Research Ethics Committee of Nanjing Drum Tower Hospital (Approval No. 2016-113-01).

The inclusion criteria for this study were defined as follows (1): maternal age ≥18 years (2); singleton pregnancy; (3) confirmed fetal viability at 11–13 GW; and (4) provision of written informed consent. The exclusion criteria were: (1) multiple pregnancy; (2) presence of major fetal structural abnormalities detected at 11–13 GW; (3) planned termination of pregnancy; and (4) cognitive impairment or inability to provide informed consent.

### 2.2 The measurement of Placental Growth Factor levels in serum

Blood samples were collected from all participants on the day of enrollment, between the 11–14 GW. In addition, for the first 1,800 participants, additional blood samples were also obtained at three subsequent time points: 18–24 GW, 28–34 GW, and after 35 GW. Serum separation was performed according to a standardized operating procedure (18). PIGF concentrations were quantified using the Cobas e602 analyzer (Roche Diagnostics, Germany). Quality control procedures adhered to both institutional and manufacturer guidelines. Specifically, the coefficient of variation for quality control materials at different concentrations within each batch was required to remain below 5%. Furthermore, quality control measurement values for each assay were required to fall within ±2 standard deviations of the established target values.

### 2.3 Adverse pregnancy outcomes

Adverse pregnancy outcomes assessed in this cohort included gestational diabetes mellitus (GDM), gestational hypertension, PE, ectopic pregnancy, placental abruption, premature rupture of membranes (PROM), spontaneous abortion, placenta praevia, single live birth, large for gestational age (LGA), SGA, and preterm birth. The definitions and diagnostic criteria for each outcome are provided in **Supplementary Material 1**. Specifically, the definitions of LGA and SGA were based on gestational age-specific growth curves constructed from our own Chinese cohort, ensuring that the cutoff values were tailored to the study population (19).

### 2.4 Covariates

Maternal covariates included maternal age (years), pre-pregnancy body mass index (BMI, kg/m²), mean arterial pressure (MAP, mmHg) measured at 11–14 GW, gestational age at the time of PIGF testing, parity (0, 1, 2, or 3), smoking status (no/yes), and medical history including diabetes (no/yes), hypertension (no/yes), renal disease (no/yes), and systemic lupus erythematosus (no/yes).

### 2.5 Statistical analysis

All statistical analyses were conducted using R software (version 4.2.2). For continuous data, descriptive statistics were expressed as mean and standard deviation. Comparisons between groups were performed using independent-sample t-tests or non-parametric tests, as appropriate. Categorical data were presented using frequency and percentage, and comparisons between groups were made using Chi-square tests or Fisher's exact test. A two-sided *P* < 0.05 was considered statistically significant. Logistic regression models were applied to assess the relationship between PIGF levels and adverse pregnancy outcomes. In the multivariable models, key covariates such as maternal age, pre-pregnancy BMI, and MAP were adjusted. Additional covariates, including parity, smoking status, and medical history of diabetes, hypertension, renal disease, and systemic lupus erythematosus, were included in sensitivity analyses. PIGF concentrations were converted to MoM, calculated by dividing the observed value by the expected median value for the corresponding gestational age. The methodology for MoM calculation was based on the approach described by H N Madsen (20)., and PIGF MoM values were obtained using calculators provided by the Fetal Medicine Foundation (https://fetalmedicine.org/). To assess potential non-linear dose–response relationships between PIGF levels and maternal-fetal outcomes, restricted cubic splines (RCS) were fitted using the R package ‘rcssci’.

## 3 Results

### 3.1 General characteristics of cohort participants

**Figure 1:**
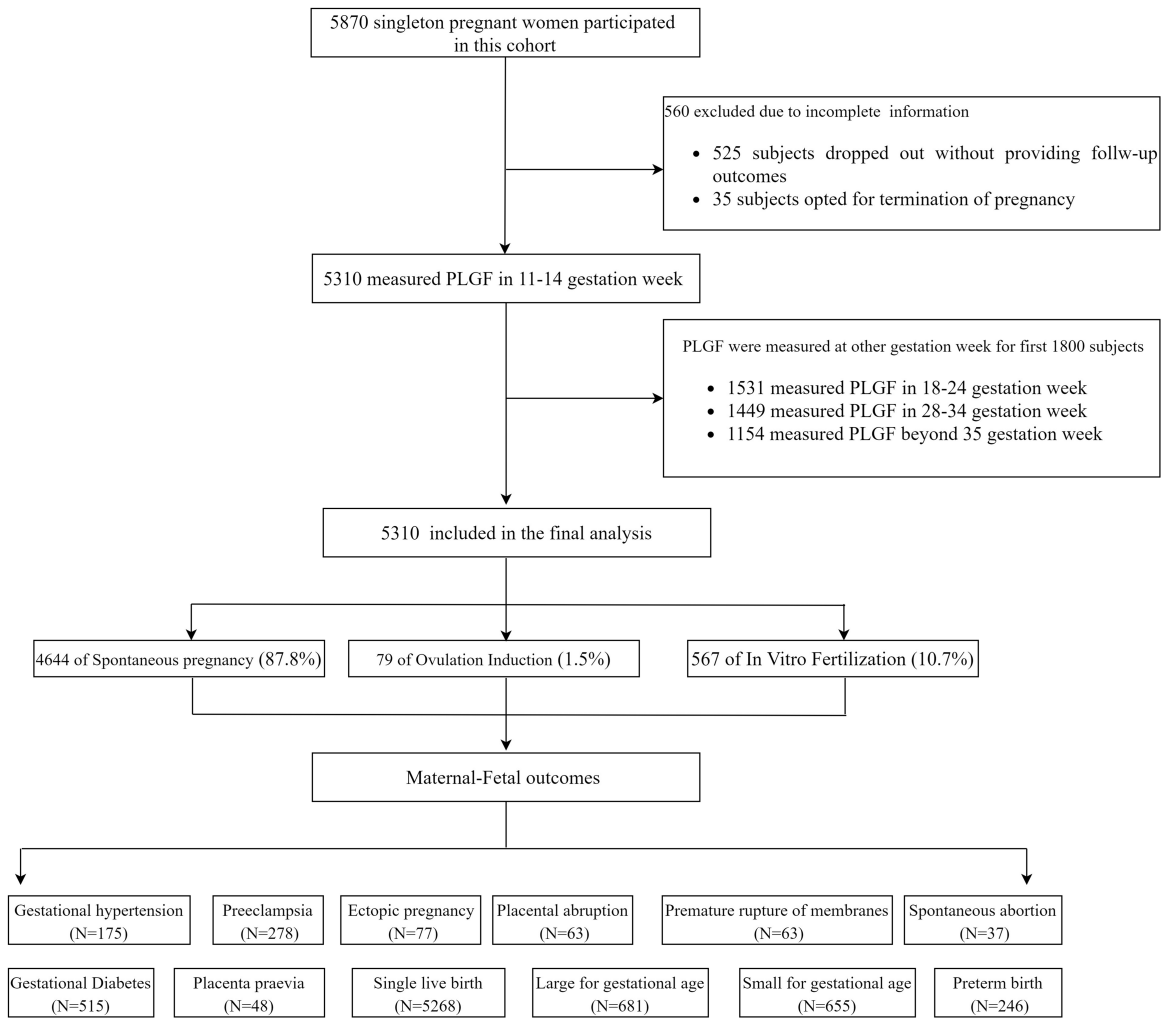
The flow diagram of the cohort study.

The flow diagram of the cohort study is shown in **Figure 1**. A total of 5,870 eligible pregnant women were initially enrolled. Among them, 560 participants (9.5%) were excluded, including 525 who discontinued participation without providing follow-up outcomes and 35 who selected to terminate the pregnancy before 28 GW. The final analytical cohort included 5,310 women: 4,664 (87.8%) in the spontaneous conception group, 79 (1.5%) in the OI group, and 567 (10.7%) in the IVF group. Follow-up results revealed that 515 participants (9.7%) developed gestational diabetes, and 278 (5.27%) were diagnosed with PE, including 64 cases (1.22%) of preterm PE. There were 5,268 singleton live births (99.23%), of which 246 (4.67%) were preterm births. A total of 655 neonates (12.43%) were SGA below the 10th percentile (SGA <10th), among them 238 cases (4.52%) classified as SGA <3rd percentile.

Baseline characteristics of participants are summarized in **Table 1**. Women in the IVF group were older and had higher pre-pregnancy body weight and MAP compared with those in the spontaneous and OI groups (*P* < 0.05), and higher incidence of gestational diabetes, PE, placental abruption, placenta praevia, and ectopic pregnancy (*P* < 0.05). Fetal outcomes were generally comparable across the groups, with no statistically significant differences observed.

**Table 1:** Study population characteristics.

Characteristic	Spontaneous (N = 4664)	OI (79)	IVF (567)	All (N=5310)	*P*
Maternal characteristic					
Maternal age, year*	29.90 ± 3.69	29.81 ± 3.03	32.45 ± 3.85	30.17 ± 3.78	<.001
Height, cm*	162.10 ± 4.70	160.62 ± 5.21	160.89 ± 5.13	161.95 ± 4.78	<.001
Pre-pregnancy weight, kg*	57.31 ± 8.77	57.72 ± 7.87	58.74 ± 9.17	57.47 ± 8.81	0.001
Pre-pregnancy Body Mass Index, kg/m2*	21.79 ± 3.06	22.39 ± 2.94	22.66 ± 3.20	21.89 ± 3.08	<.001
Early pregnancy mean arterial pressure, mmHg*	83.07 ± 7.90	83.52 ± 7.11	86.08 ± 8.51	83.39 ± 8.01	<.001
Placental growth factor, pg/mL*	32.51 ± 46.69	37.35 ± 50.67	30.61 ± 16.48	32.38 ± 44.52	0.382
Gestational week at Delivery, week*	38.95 ± 2.14	38.82 ± 2.44	38.60 ± 2.35	38.91 ± 2.17	<.001
Maternal outcome					
Gestational Diabetes, n (%)	405(8.68)	7(8.86)	103(18.17)	515(9.70)	<.001
Gestational hypertension, n (%)	145(3.11)	2(2.53)	28(4.94)	175(3.30)	0.065
Preeclampsia, n (%)	215(4.64)	6(7.69)	57(10.16)	278(5.27)	<.001
Preterm Preeclampsia, n (%)	53(1.14)	1(1.28)	10(1.79)	64(1.22)	0.425
Ectopic pregnancy, n (%)	38(0.81)	2(2.53)	37(6.53)	77(1.45)	<.001
Placental Abruption, n (%)	49(1.05)	0(0.00)	14(2.47)	63(1.19)	0.020
Premature rupture of membranes, n(%)	996(21.36)	16(20.25)	104(18.34)	1116(21.02)	0.247
Spontaneous abortion, n (%)	30(0.64)	1(1.27)	6(1.06)	37(0.70)	0.225
Placenta praevia, n (%)	31(0.66)	1(1.27)	16(2.82)	48(0.90)	<.001
Fetal outcome					
Single live birth, n (%)	4630(99.29)	78(98.73)	560(98.77)	5268(99.23)	0.256
NICU > 24h, n (%)	9(0.19)	0(0.00)	0(0.00)	9(0.17)	0.659
Neonatal asphyxia, n (%)	3(0.06)	0(0.00)	0(0.00)	3(0.05)	1.000
Large for gestational age, n (%)	591(12.73)	83(14.77)	7(8.97)	681(12.73)	0.230
SGA < 10th, n(%)	577(12.46)	7(8.97)	71(12.66)	655(12.43)	0.642
SGA < 3rd, n (%)	202(4.36)	3(3.85)	33(5.88)	238(4.52)	0.251
Preterm birth (< 37w), n (%)	209(4.51)	3(3.85)	34(6.07)	246(4.67)	0.241

*Data are presented as mean ± standard deviation. Spontaneous, Spontaneous pregnancy; OI, Ovulation Induction; IVF, *In Vitro* Fertilization; SGA < 10th, birth weight below the 10th percentile for gestational age; SGA < 3rd, birth weight below the 3rd percentile for gestational age.

### 3.2 Association between PIGF levels and adverse maternal-fetal outcomes

**Table 2** summarizes the associations between serum PIGF levels in 11–14 GW and adverse pregnancy outcomes. In adjusted logistic regression models, higher PIGF concentrations were inversely associated with PE (odds ratio [OR] = 0.97, 95% confidence interval [CI]: 0.96 – 0.98), preterm PE (OR = 0.96, 95% CI: 0.94 – 0.98), SGA <10th percentile (OR = 0.99, 95% CI: 0.98 – 0.99), and SGA <3rd percentile (OR = 0.98, 95% CI: 0.97 – 0.99). When using PIGF MoM values, the inverse associations were stronger for PE (OR = 0.32, 95% CI: 0.21 – 0.48), preterm PE (OR = 0.23, 95% CI: 0.09 – 0.56), SGA <10th percentile (OR = 0.67, 95% CI: 0.54 – 0.83), and SGA <3rd percentile (OR = 0.43, 95% CI: 0.29 – 0.64). These associations remained robust in sensitivity analyses further adjusting for parity, smoking, history of diabetes, history of hypertension, history of renal disease, and systemic lupus erythematosus (**Supplementary Table S1**).

**Table 2:** Multivariable logistic regression analysis on the association between PIGF and adverse pregnancy outcomes.

Outcome	PIGF			Mom value of PIGF		
	OR*	95%CI	*P*	OR *	95%CI	*P*
Maternal outcome						
Gestational Diabetes	1.00	0.99-1.01	0.908	1.01	0.92-1.21	0.436
Gestational hypertension	0.99	0.98-1.00	0.883	0.94	0.70-1.26	0.683
Preeclampsia	0.97	0.96-0.98	<0.001	0.32	0.21-0.48	<0.001
Preterm Preeclampsia	0.96	0.94-0.98	0.001	0.23	0.09-0.56	0.001
Ectopic pregnancy	0.99	0.98-1.01	0.654	1.00	0.69-1.46	0.985
Placental Abruption	0.99	0.98-1.01	0.501	0.83	0.46-1.47	0.513
Premature rupture of membranes	1.00	0.98-1.00	0.355	0.95	0.85-1.06	0.372
Spontaneous abortion	1.00	0.98-1.02	0.824	0.92	0.48-1.77	0.805
Placenta praevia	0.98	0.96-1.01	0.144	0.62	0.28-1.38	0.246
Fetal outcome						
Single live birth	1.00	0.98-1.02	0.660	1.18	0.58-2.39	0.648
Large for gestational age	1.00	0.99-1.01	0.839	1.08	0.96-1.21	0.220
SGA < 10th	0.99	0.98-0.99	<0.001	0.67	0.54-0.83	<0.001
SGA < 3rd	0.98	0.97-0.99	<0.001	0.43	0.29-0.64	<0.001
Preterm birth	1.00	0.99-1.01	0.805	1.01	0.82-1.25	0.928

OR, odds ratio; CI, confidence interval. SGA < 10th, birth weight below the 10th percentile for gestational age; SGA < 3rd, birth weight below the 3rd percentile for gestational age.

*adjusted model: adjusted for maternal age, BMI, mean arterial pressure, gestational week for PIGF testing.

### 3.3 Subgroup analysis

Subgroup analysis stratified by mode of conception are presented in **Table 3**. In the spontaneous conception group, serum PIGF concentrations were inversely related to PE (OR = 0.97, 95% CI: 0.96 – 0.98), preterm PE (OR = 0.96, 95% CI: 0.94 – 0.99), SGA <10th percentile (OR = 0.99, 95% CI: 0.98 – 0.99), and SGA <3rd percentile (OR = 0.98, 95% CI: 0.97 – 0.99). Similarly, inverse associations between PIGF levels and PE were observed in both the IVF and OI subgroups. In these groups, the ORs for PE were 0.95 (95% CI: 0.92 – 0.97) for raw PIGF levels and 0.14 (95% CI: 0.05 – 0.37) for PIGF MoM values, respectively. However, no significant associations were found between PIGF levels (both raw and MoM values) and the risks of preterm PE, SGA <10th percentile, or SGA <3rd percentile in the IVF and OI subgroups. Interaction analysis with GDM (**Supplementary Table S2**) showed that the associations between PIGF levels and PE were evident only in the non-GDM group, whereas no significant associations were observed in the GDM group. By contrast, PIGF was significantly inversely associated with SGA in both the GDM and non-GDM groups. The inverse association of PIGF MoM values with SGA <3rd percentile was stronger in the GDM group (OR = 0.24, 95% CI: 0.06 – 0.86), while it also remained significant in the non-GDM group (OR = 0.46, 95% CI: 0.30 – 0.69).

**Table 3:** Subgroup analysis on the association between PIGF level and adverse pregnancy outcomes.

Outcome	Spontaneous			IVF and OI		
	OR*	95%CI	*P*	OR*	95%CI	*P*
PIGF						
Preeclampsia	0.97	0.96-0.98	<0.001	0.95	0.92-0.97	0.001
Preterm Preeclampsia	0.96	0.94-0.99	0.003	0.96	0.91-1.02	0.220
SGA<10th	0.99	0.98-0.99	0.001	0.99	0.97-1.01	0.177
SGA<3rd	0.98	0.97-0.99	<0.001	0.99	0.97-1.02	0.596
MoM value of PIGF level						
Preeclampsia	0.36	0.23-0.58	<0.001	0.13	0.05-0.37	<0.001
Preterm Preeclampsia	0.24	0.09-0.62	0.003	0.27	0.03-2.60	0.256
SGA<10th	0.67	0.54-0.84	<0.001	0.63	0.33-1.22	0.170
SGA<3rd	0.37	0.24-0.58	<0.001	0.82	0.37-1.83	0.631

Spontaneous, Spontaneous pregnancy; OI, Ovulation Induction; IVF, *In Vitro* Fertilization; OR, odds ratio; CI, confidence interval. SGA<10th, birth weight below the 10th percentile for gestational age; SGA<3rd, birth weight below the 3rd percentile for gestational age. *adjusted model: adjusted for maternal age, BMI, mean arterial pressure, gestational week for PIGF testing.

### 3.4 Evaluation of predictive performance

The predictive performance of PIGF levels in the 11–14 GW for identifying adverse pregnancy outcomes is detailed in **Table 4**. Generally, PIGF levels during early pregnancy showed an area under the curve (AUC) of 0.61 for predicting PE, 0.64 for preterm PE, 0.57 for SGA < 10th percentile, and 0.59 for SGA < 3rd percentile. Similar trends were observed in the spontaneous conception subgroup (N = 4635). In contrast, within the IVF and OI subgroup (N = 639), no statistically significant associations were identified between PIGF levels and the risks of preterm PE, SGA <10th percentile, or SGA <3rd percentile. Nonetheless, for PE in the IVF and OI subgroup, the AUC reached 0.65, with a sensitivity of 0.78 and a specificity of 0.49. The MoM values of PIGF displayed similar predictive performance to that of raw PIGF levels.

**Table 4:** Predictive performance of the PIGF levels at 11–14 gestational week for the detection of adverse pregnancy outcomes.

Group	Outcome	PIGF			Mom value of PIGF		
		AUC (95%CI)	Se (95%CI)	Sp (95%CI)	AUC (95%CI)	Se (95%CI)	Sp (95%CI)
All (N = 5310)	PE	0.61 (0.60-0.63)	0.70 (0.64 – 0.76)	0.47 (0.45 – 0.48)	0.61 (0.59-0.62)	0.60 (0.54 – 0.66)	0.57 (0.56 – 0.59)
	Preterm PE	0.64 (0.63-0.65)	0.55 (0.42 – 0.67)	0.68 (0.67 – 0.70)	0.63 (0.62-0.65)	0.53 (0.40 – 0.66)	0.70 (0.69 – 0.71)
	SGA<10^th^	0.57 (0.56-0.58)	0.48 (0.45 – 0.52)	0.64 (0.62 – 0.65)	0.57 (0.56-0.59)	0.47 (0.43 – 0.51)	0.65 (0.64 – 0.67)
	SGA<3^rd^	0.59 (0.58-0.61)	0.53 (0.46 – 0.59)	0.62 (0.61 – 0.64)	0.60 (0.59-0.62)	0.53 (0.46 – 0.59)	0.65 (0.63 – 0.66)
Spontaneous Pregnancy (N = 4635)	PE	0.60 (0.59-0.62)	0.87 (0.82 – 0.92)	0.27 (0.26 – 0.29)	0.61 (0.60-0.63)	0.70 (0.64 – 0.76)	0.47 (0.45 – 0.48)
	Preterm PE	0.64 (0.62-0.65)	0.53 (0.39 – 0.67)	0.69 (0.68 – 0.70)	0.62 (0.61-0.64)	0.45 (0.32 – 0.59)	0.76 (0.75 – 0.77)
	SGA<10^th^	0.57 (0.56-0.58)	0.48 (0.44 – 0.53)	0.64 (0.63 – 0.66)	0.57 (0.56-0.59)	0.44 (0.40 – 0.48)	0.69 (0.67 – 0.70)
	SGA<3^rd^	0.60 (0.59-0.62)	0.54 (0.46 – 0.61)	0.63 (0.61 – 0.64)	0.61 (0.60-0.63)	0.42 (0.35 – 0.49)	0.76 (0.75 – 0.78)
IVF and OI* (N = 639)	PE	0.65 (0.61-0.68)	0.78 (0.66 – 0.87)	0.49 (0.45 – 0.54)	0.66 (0.63-0.70)	0.67 (0.54 – 0.78)	0.63 (0.59 – 0.69)

AU, Area Under the Curve; Se, Sensitivity; Sp, Specificity; PE, Preeclampsia; SGA,C Small for gestational age; CI, Confidence Interval; Spontaneous, Spontaneous pregnancy; OI, Ovulation Induction; IVF, *In Vitro* Fertilization. *In the IVF and OI subgroup, no significant statistical association was identified between PIGF levels and the outcomes of preterm PE, SGA<10th and SGA<3rd. hence no predictive performance analysis is performed here.

To place our findings in context, we further summarized recent studies published in the past five years that evaluated PIGF for risk stratification across different pregnancy complications (**Supplementary Table S3**). These studies covered diverse clinical indications including preterm birth, PE, discordant fetal growth, and ectopic pregnancy, with reported PIGF thresholds ranging from 15.5 pg/ml to 290 pg/ml. The predictive performance varied by outcome and study design (with AUCs ranging from 0.72 to 0.90), but consistently supported the potential clinical utility of PIGF as a biomarker for early risk stratification in pregnancy.

### 3.5 Dose-response relationship analysis

**Figure 2:**
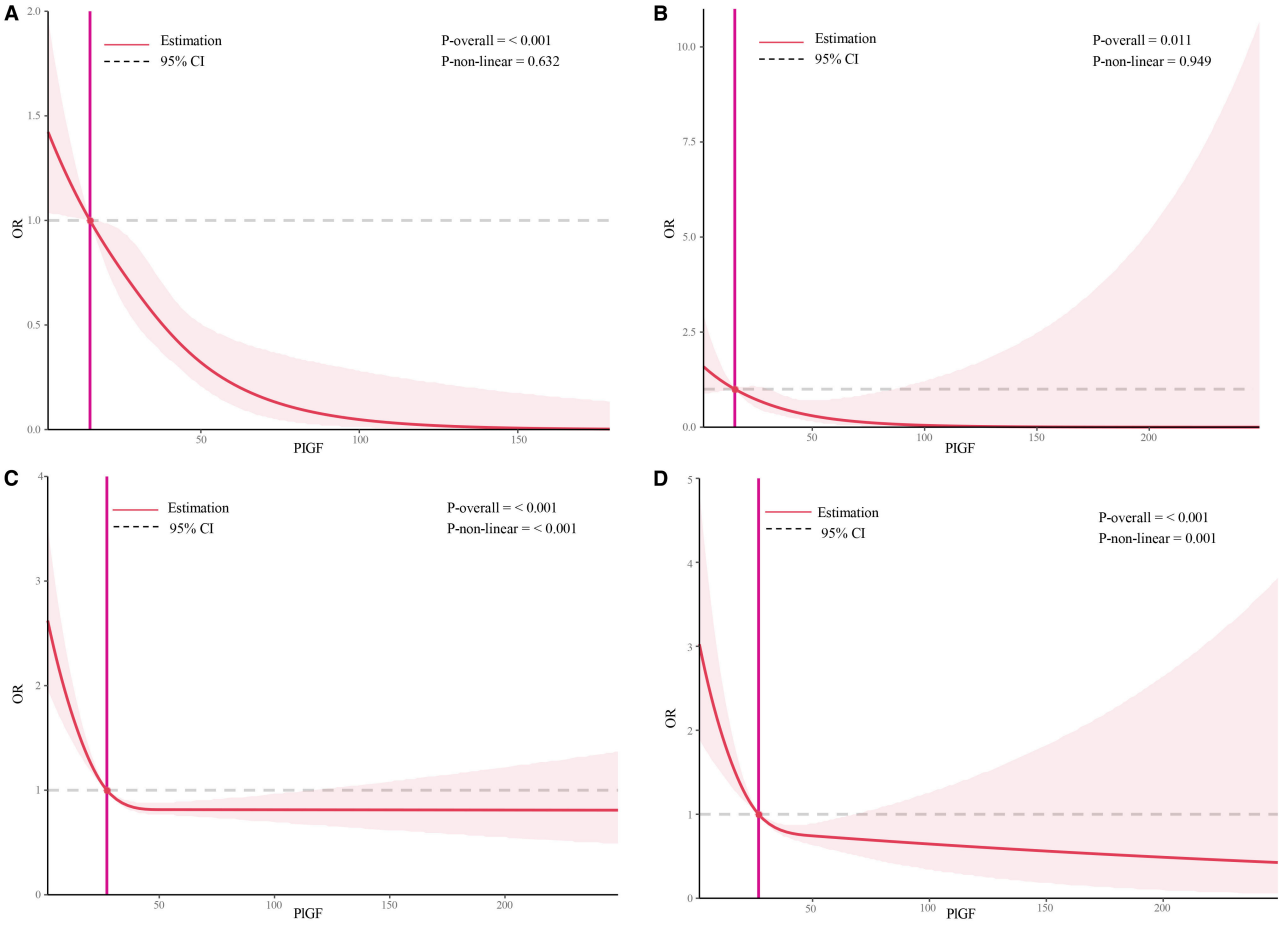
Restricted cubic spline plots of the association between serum placental growth factor level and adverse pregnancy outcomes. **(A)** Restricted cubic spline analysis of PLGF in relation to PE outcomes, with a simplified plot using an odds ratio of 1 as the cutoff point; **(B)** Restricted cubic spline analysis of PLGF in relation to preterm PE outcomes, with a simplified plot using an odds ratio of 1 as the cutoff point; **(C)** Restricted cubic spline analysis of PLGF in relation to SGA<10th, with a simplified plot using an odds ratio of 1 as the cutoff point; **(D)** Restricted cubic spline analysis of PLGF in relation to SGA<3rd, with a simplified plot using an odds ratio of 1 as the cutoff point.

The dose–response relationships between serum PIGF concentrations measured during 11–14 GW and adverse pregnancy outcomes were examined using RCS models (**Figure 2**). A linear inverse association was observed for both PE (**Figure 2A**) and preterm PE (**Figure 2B**). In contrast, non-linear associations were found for SGA <10th percentile and SGA <3rd percentile (**Figures 2C, D**, respectively). The optimal PIGF cut-off value for minimizing the risk of SGA <10th percentile was 27.27 pg/mL, while that for SGA <3rd percentile was 26.92 pg/mL (**Table 5**).

**Table 5:** Threshold effect analysis of PIGF level on both SGA<10th and SGA<3rd by the two-piecewise logistic regression.

Inflection point	Unadjusted model			Adjusted model*		
	OR	95%CI	*P*	OR	95%CI	*P*
SGA<10th						
PIGF ≤27.267	0.96	0.94-0.98	<0.001	0.96	0.94-0.98	<0.001
PIGF >27.267	1.00	0.99-1.01	0.846	1.00	0.99-1.01	0.828
MoM values of PIGF ≤0.716	0.22	0.11-0.45	<0.001	0.22	0.11-0.45	<0.001
MoM values of PIGF >0.716	0.98	0.82-1.17	0.826	0.98	0.81-1.17	0.795
SGA<3rd						
PIGF ≤26.919	0.95	0.92-0.98	<0.001	0.95	0.92-0.98	0.001
PIGF >26.919	0.99	0.98-1.01	0.465	0.99	0.98-1.01	0.378
MoM values of PIGF ≤0.713	0.15	0.05-0.42	<0.001	0.16	0.05-0.45	<0.001
MoM values of PIGF >0.713	0.77	0.47-1.29	0.333	0.76	0.45-1.30	0.322

SGA, Small for gestational age; OR, odds ratio; CI, confidence interval. *adjusted model: adjusted for maternal age, BMI, mean arterial pressure, gestational week for PIGF testing.

Two-piecewise logistic regression models were further used to evaluate the threshold effects of PIGF on SGA outcomes, as shown in **Table 4**. After adjusting for potential confounders, a significant inverse association was observed below the inflection points: for SGA <10th percentile (OR = 0.96, 95% CI: 0.94 – 0.98) and SGA <3rd percentile (OR = 0.95, 95% CI: 0.92 – 0.98). No significant associations were found to the above the thresholds (*P* > 0.05).

### 3.6 Comparative analysis of the association between PIGF levels and adverse maternal-fetal outcomes across gestational stages

The results of a logistic regression analysis evaluating the association between PIGF levels and adverse maternal-fetal outcomes at different stages of pregnancy are presented in **Supplementary Table S4**. Overall, PIGF MoM values were consistently inversely associated with PE, SGA <10th percentile, and SGA <3rd percentile across all gestational stages. Specifically, for PE, the ORs were 0.27 (95% CI: 0.14 – 0.55) at 18–24 GW, 0.11 (95% CI: 0.04 – 0.27) at 28–34 GW, and 0.18 (95% CI: 0.06 – 0.49) after 35 GW. Additionally, PIGF MoM values during 28–34 GW were inversely associated with preterm birth (OR = 0.62, 95% CI: 0.41 – 0.93) and positively associated with LGA (OR = 1.39, 95% CI: 1.21 – 1.61). Detailed characteristics of the study population across different gestational stages are provided in **Supplementary Table S5**.

The predictive performance of PIGF levels for adverse pregnancy outcomes at different gestational stages is summarized in **Table 6**. The highest predictive performance for both PE and preterm PE was observed during 28–34 GW, with AUCs of 0.79 (95% CI: 0.77 – 0.81) and 0.86 (95% CI: 0.85 – 0.88), respectively. Similarly, the best performance for SGA <10th percentile was also noted during 28–34 GW, with an AUC of 0.63 (95% CI: 0.57 – 0.66). In contrast, the optimal predictive performance for SGA <3rd percentile was observed after 35 GW, with an AUC of 0.69 (95% CI: 0.66 – 0.72). Moreover, PIGF levels during 28–34 GW demonstrated predictive value for LGA (AUC = 0.58, 95% CI: 0.56 – 0.61) and preterm birth (AUC = 0.65, 95% CI: 0.62 – 0.67), as shown in **Supplementary Table S6**.

**Table 6:** The predictive performance of PIGF values for adverse pregnancy outcomes at different stages of pregnancy.

GW	Outcome	PIGF			MOM of PIGF		
		AUC (95%CI)	Se (95%CI)	Sp (95%CI)	AUC (95%CI)	Se (95%CI)	Sp (95%CI)
11–14 GW (N = 5310)	PE	0.61 (0.60-0.63)	0.70 (0.64-0.76)	0.47 (0.45-0.48)	0.61 (0.59-0.62)	0.60 (0.54-0.66)	0.58 (0.56-0.59)
	Preterm PE	0.64 (0.63-0.65)	0.55 (0.42-0.67)	0.69 (0.67-0.70)	0.63 (0.62-0.64)	0.53 (0.40-0.66)	0.70 (0.69-0.71)
	SGA < 10th	0.57 (0.56-0.58)	0.48 (0.45-0.52)	0.64 (0.62-0.65)	0.57 (0.56-0.59)	0.47 (0.43-0.51)	0.65 (0.64-0.67)
	SGA < 3rd	0.59 (0.58-0.61)	0.53 (0.46-0.59)	0.62 (0.61-0.64)	0.60 (0.59-0.62)	0.53 (0.46-0.59)	0.65 (0.63-0.66)
18–24 GW (N = 1531)	PE	0.70 (0.68-0.73)	0.54 (0.41-0.67)	0.79 (0.77-0.81)	0.66 (0.63-0.68)	0.68 (0.54-0.79)	0.62 (0.59-0.65)
	Preterm PE	0.73 (0.71-0.75)	0.67 (0.43-0.85)	0.72 (0.70-0.74)	0.66 (0.63-0.68)	0.57 (0.34-78.2)	0.73 (0.71-0.75)
	SGA < 10th	0.61 (0.58-0.63)	0.43 (0.36-0.50)	0.74 (0.72-0.77)	0.61 (0.58-0.63)	0.47 (0.40-0.55)	0.71 (0.68-0.73)
	SGA < 3rd	0.65 (0.62-0.67)	0.51 (0.39-0.64)	0.74 (0.72-0.76)	0.66 (0.64-0.68)	0.73 (0.60-0.82)	0.55 (0.52-0.57)
28–34 GW (N = 1449)	PE	0.79 (0.77-0.81)	0.64 (0.50-0.77)	0.82 (0.80-0.84)	0.78 (0.76-0.80)	0.69 (0.53-0.80)	0.78 (0.76-0.80)
	Preterm PE	0.86 (0.85-0.88)	0.87 (0.62-0.98)	0.81 (0.79-0.83)	0.85 (0.83-0.87)	0.87 (0.62-0.98)	0.81 (0.80-0.84)
	SGA < 10th	0.63 (0.57-0.66)	0.57 (0.49-0.65)	0.63 (0.60-0.66)	0.65 (0.62-0.67)	0.61 (0.53-0.68)	0.61 (0.58-0.63)
	SGA < 3rd	0.67 (0.65-0.70)	0.64 (0.51-0.76)	0.62 (0.60-0.65)	0.69 (0.67-0.72)	0.69 (0.56-0.80)	0.60 (0.57-0.62)
>35 GW (N = 1154)	PE	0.74 (0.71-0.77)	0.73 (0.54-0.88)	0.65 (0.62-0.68)	0.72 (0.70-0.75)	0.83 (0.65-0.94)	0.53(0.50-0.56)
	Preterm PE*	–	–	–	–	–	–
	SGA < 10th	0.61 (0.58-0.64)	0.64 (0.55-0.72)	0.57 (0.54-0.60)	0.61 (0.58-0.64)	0.67 (0.58-0.75)	0.54(0.51-0.57)
	SGA < 3rd	0.69 (0.67-0.72)	0.55 (0.40-0.69)	0.76 (0.74-0.79)	0.69 (0.66-0.72)	0.53 (0.38-0.67)	0.77 (0.74-0.79)

PIGF, Placental Growth Factor; AUC, Area Under the Curve; Se, Sensitivity; Sp, Specificity; CI, Confidence Interval; GW, Gestational Weeks; PE, Preeclampsia; SGA < 10th, birth weight below the 10th percentile for gestational age; SGA < 3rd, birth weight below the 3rd percentile for gestational age.

*Preterm PE is defined as occurring before 37 GW. Consequently, in cases extending beyond 35 GW, preterm PE would have manifested and been addressed, resulting in the absence of data for this group.

## 4 Discussion

In this prospective cohort study, we investigated the associations between maternal serum PIGF concentrations measured at 11–14 GW and a range of adverse maternal and fetal outcomes. Both raw PIGF values and MoM values showed inverse associations with PE, preterm PE, and SGA below the 10th and 3rd percentiles. These associations were consistent across different modes of conception, with PIGF showing predictive value for PE regardless of whether the pregnancy was spontaneous, achieved through OI or IVF. The highest predictive performance for PE was observed during the 28–34 GW. Moreover, dose-response analysis suggested a linear inverse association between PIGF and PE outcomes, whereas the associations with SGA <10th and <3rd percentiles were non-linear, indicating a possible threshold effect.

PIGF, an angiogenic factor, has been reported to be associated with pregnancy complications, particularly PE and SGA. However, existing literature reports inconsistent findings regarding its predictive performance for PE (15). Some researchers showed that PIGF had relatively high predictive accuracy for PE at 11–14 GW, with AUCs above 0.7 and sensitivities above 60% (21, 22), and others showed its limited predictive performance, with AUCs below 0.6 and sensitivities below 25% (23, 24). These discrepancies may stem from differences in study populations, cutoff thresholds, or analytical platforms used. In our cohort, the predictive performance of PIGF for PE at 11–14 GW was moderate (AUC = 0.61; sensitivity = 70%), suggesting that although PIGF contributes to risk stratification, it may not serve as a standalone predictor, and PIGF with maternal demographic characteristics, MAP, uterine artery pulsatility index, and pregnancy-associated plasma protein A could significantly improve predictive performance for PE at 11–14 GW (25). Consistently, the Fetal Medicine Foundation (FMF) Bayes-based competing risk model and other studies have also recommended integrating PIGF with maternal characteristics and additional biomarkers in the first trimester to optimize PE prediction (26–29). Similarly, the predictive performance for SGA <10th and <3rd percentiles was limited when using PIGF alone, reinforcing the need to integrate PIGF with other clinical and ultrasonography markers to enhance screening accuracy. In addition, our findings revealed that PIGF MoM values showed stronger associations with PE and SGA than absolute concentrations, which underscores the importance of standardizing for gestational age of and maternal characteristics (30, 31).

Previous studies have provided limited data regarding the influence of conception method on PIGF levels. A study reported no significant difference in serum PIGF concentrations between IVF and spontaneous pregnancies at 10 weeks’ gestation (32), a finding consistent with our results. In the present analysis, the predictive performance of PIGF for PE was slightly higher in the IVF and OI subgroups than in the spontaneous conception group. However, no significant associations were found between PIGF levels and preterm PE or SGA outcomes in the IVF and OI subgroups. These findings suggest that while PIGF retains its association with PE across conception methods, its role in predicting other outcomes may be more variable. This highlights the potential need for tailored screening strategies when evaluating pregnancy risks in assisted reproductive technology (ART) populations. Notably, in our interaction analysis with GDM, the inverse association between maternal PIGF levels and the risk of SGA was more pronounced in the GDM group than in the non-GDM group, although the underlying mechanisms and potential explanations remain to be elucidated.

This study also explored the dose-response relationship between early pregnancy PIGF levels and adverse outcomes, with a particular focus on non-linear associations. RCS analyses demonstrated linear associations between PIGF and both PE and preterm PE. In contrast, the relationships between PIGF and SGA <10th or <3rd percentile were nonlinear. The strongest associations for SGA were observed below the identified inflection points (27.3 pg/mL for SGA <10th and 26.9 pg/mL for SGA <3rd), while associations diminished beyond these thresholds. These findings are consistent with prior reports (33), which also reported a nonlinear association between mid-pregnancy PIGF and SGA. RCS modeling, with its ability to flexibly capture inflection points, provides valuable insight for determining clinically meaningful thresholds that may optimize screening and intervention strategies.

This study has several notable strengths. First, it utilized a large, prospective cohort with detailed clinical data and well-defined pregnancy outcomes, allowing for comprehensive and reliable analyses. Second, the inclusion of participants with different modes of conception—spontaneous, OI, and IVF—enabled stratified subgroup analyses that are rarely explored in prior research. Third, we assessed the predictive performance of PIGF across different gestational windows, identifying that its predictive ability for PE peaked at 28–34 GW, which may inform the optimal timing for clinical screening.

However, several limitations should be acknowledged. Although the prospective design strengthens the temporal relationship between exposure and outcome, the observational nature of the study does not permit causal inferences. Despite adjustment for a range of maternal characteristics and clinical factors, residual confounding from unmeasured variables may still exist. Additionally, subgroup analyses in the IVF and OI populations were limited by smaller sample sizes, potentially reducing statistical power to detect associations with outcomes such as preterm PE or SGA. Finally, the cohort was drawn from a single regional center, which may limit the generalizability of our findings to broader populations or healthcare systems. Despite these limitations, this study adds important evidence on the association between early pregnancy PIGF levels and adverse outcomes, including nuanced subgroup differences by conception mode and non-linear dose-response patterns with SGA. Future studies should aim to track dynamic changes in PIGF throughout gestation and evaluate whether incorporating PIGF into multi-marker screening algorithms can improve early risk stratification and guide targeted interventions.

In conclusion, our study demonstrates that maternal serum PIGF levels, particularly MoM-standardized values, are significantly associated with the risk of PE and SGA, especially when measured between 28–34 GW. The predictive value of PIGF varies by gestational age and conception mode, with the strongest performance observed in spontaneous pregnancies during mid-to-late gestation. Moreover, a non-linear dose–response relationship was observed between PIGF and SGA risk, suggesting a threshold effect. These findings underscore the potential of gestational age–tailored PIGF screening for pregnancy risk stratification, and highlight the need for further validation in multi-center studies with diverse populations.

## Data Availability

The raw data supporting the conclusions of this article will be made available by the authors, without undue reservation.

## Supplementary material

Supplementary Material 1 The diagnostic criteria and definitions of study outcome.

## References

[1] Crump C Sundquist J Sundquist K. Adverse pregnancy outcomes and long-term risk of chronic kidney disease in women: national cohort and co-sibling study. Am J obstetrics gynecology. (2023) 230:563.e1–563.e20. doi: 10.1016/j.ajog.2023.10.008, PMID: 37827269 PMC11006822

[2] Crump C Sundquist J McLaughlin MA Dolan SM Govindarajulu U Sieh W. Adverse pregnancy outcomes and long term risk of ischemic heart disease in mothers: national cohort and co-sibling study. BMJ. (2023) 380:e072112. doi: 10.1136/bmj-2022-072112, PMID: 36724989 PMC9890184

[3] Khan SS Petito LC Huang X Harrington K McNeil RB Bello NA. Body mass index, adverse pregnancy outcomes, and cardiovascular disease risk. Circ Res. (2023) 133:725–35. doi: 10.1161/CIRCRESAHA.123.322762, PMID: 37814889 PMC10578703

[4] Patro Golab B Santos S Voerman E Lawlor DA Jaddoe VWV Gaillard R. Influence of maternal obesity on the association between common pregnancy complications and risk of childhood obesity: an individual participant data meta-analysis. Lancet Child Adolesc Health. (2018) 2:812–21. doi: 10.1016/S2352-4642(18)30273-6, PMID: 30201470 PMC6196075

[5] Singh M Wambua S Lee SI Okoth K Wang Z Fazla F. Autoimmune diseases and adverse pregnancy outcomes: an umbrella review. Lancet. (2023) 402 Suppl 1:S84. doi: 10.1016/S0140-6736(23)02128-1, PMID: 37997130

[6] Xiong Y Wang J Huang S Liu C Liu Y Qi Y. Association between maternal prepregnancy body mass index and pregnancy outcomes following assisted reproductive technology: A systematic review and dose-response meta-analysis. Obes Rev. (2021) 22:e13219. doi: 10.1111/obr.13219, PMID: 33554474

[7] McLennan AS Gyamfi-Bannerman C Ananth CV Wright JD Siddiq Z D'Alton ME. The role of maternal age in twin pregnancy outcomes. Am J obstetrics gynecology. (2017) 217:80 e1– e8. doi: 10.1016/j.ajog.2017.03.002, PMID: 28286050 PMC5571734

[8] Bramham K Parnell B Nelson-Piercy C Seed PT Poston L Chappell LC. Chronic hypertension and pregnancy outcomes: systematic review and meta-analysis. BMJ. (2014) 348:g2301. doi: 10.1136/bmj.g2301, PMID: 24735917 PMC3988319

[9] De Falco S. The discovery of placenta growth factor and its biological activity. Exp Mol Med. (2012) 44:1–9. doi: 10.3858/emm.2012.44.1.025, PMID: 22228176 PMC3277892

[10] Levine RJ Maynard SE Qian C Lim KH England LJ Yu KF. Circulating angiogenic factors and the risk of preeclampsia. New Engl J Med. (2004) 350:672–83. doi: 10.1056/NEJMoa031884, PMID: 14764923

[11] Balyan K Humtso BY Meena B Sapna S Rana A Kumar M. Materno-fetal outcome with plgf above or below cutoff during second half of pregnancy in high-risk women. Int J gynaecology obstetrics: Off Organ Int Fed Gynaecology Obstetrics. (2023) 165:211–9. doi: 10.1002/ijgo.15143, PMID: 37814586

[12] Duhig KE Myers J Seed PT Sparkes J Lowe J Hunter RM. Placental growth factor testing to assess women with suspected pre-eclampsia: A multicentre, pragmatic, stepped-wedge cluster-randomised controlled trial. Lancet. (2019) 393:1807–18. doi: 10.1016/S0140-6736(18)33212-4, PMID: 30948284 PMC6497988

[13] Benton SJ McCowan LM Heazell AE Grynspan D Hutcheon JA Senger C. Placental growth factor as a marker of fetal growth restriction caused by placental dysfunction. Placenta. (2016) 42:1–8. doi: 10.1016/j.placenta.2016.03.010, PMID: 27238707

[14] Sovio U Gaccioli F Cook E Charnock-Jones DS Smith GCS. Maternal serum levels of soluble fms-like tyrosine kinase-1 and placental growth factor at 20 and 28 weeks of gestational age and the risk of spontaneous preterm birth. Am J obstetrics gynecology. (2023) 229:164 e1– e18. doi: 10.1016/j.ajog.2023.02.001, PMID: 36758709

[15] Agrawal S Shinar S Cerdeira AS Redman C Vatish M. Predictive performance of plgf (Placental growth factor) for screening preeclampsia in asymptomatic women: A systematic review and meta-analysis. Hypertension (Dallas Tex: 1979). (2019) 74:1124–35. doi: 10.1161/HYPERTENSIONAHA.119.13360, PMID: 31522621

[16] Zhang L Li W Chi X Sun Q Li Y Xing W. Predictive performance of sflt-1, plgf and the sflt-1/plgf ratio for preeclampsia: A systematic review and meta-analysis. J Gynecol Obstet Hum Reprod. (2025) 54:102925. doi: 10.1016/j.jogoh.2025.102925, PMID: 39947348

[17] Chen W Wei Q Liang Q Song S Li J. Diagnostic capacity of sflt-1/plgf ratio in fetal growth restriction: A systematic review and meta-analysis. Placenta. (2022) 127:37–42. doi: 10.1016/j.placenta.2022.07.020, PMID: 35952596

[18] Zeisler H Llurba E Chantraine F Vatish M Staff AC Sennstrom M. Predictive value of the sflt-1:Plgf ratio in women with suspected preeclampsia. New Engl J Med. (2016) 374:13–22. doi: 10.1056/NEJMoa1414838, PMID: 26735990

[19] Wang Y Wang Y Tang HR Zhang Y Dai CY Li J. Establishment method and significance of birthweight curve and reference in single center. Chin J Obstetrics Gynecology. (2023) 58:334–42. doi: 10.3760/cma.j.issn.0529-567x.2014.07.001, PMID: 37217340

[20] Madsen HN Ball S Wright D Torring N Petersen OB Nicolaides KH. A reassessment of biochemical marker distributions in trisomy 21-affected and unaffected twin pregnancies in the first trimester. Ultrasound obstetrics gynecology: Off J Int Soc Ultrasound Obstetrics Gynecology. (2011) 37:38–47. doi: 10.1002/uog.8845, PMID: 20878678

[21] Foidart JM Munaut C Chantraine F Akolekar R Nicolaides KH. Maternal plasma soluble endoglin at 11–13 weeks' Gestation in pre-eclampsia. Ultrasound obstetrics gynecology: Off J Int Soc Ultrasound Obstetrics Gynecology. (2010) 35:680–7. doi: 10.1002/uog.7621, PMID: 20205159

[22] Youssef A Righetti F Morano D Rizzo N Farina A. Uterine artery doppler and biochemical markers (Papp-a, pigf, sflt-1, P-selectin, ngal) at 11 + 0 to 13 + 6 weeks in the prediction of late (> 34 weeks) pre-eclampsia. Prenatal diagnosis. (2011) 31:1141–6. doi: 10.1002/pd.2848, PMID: 22034048

[23] Schneuer FJ Nassar N Guilbert C Tasevski V Ashton AW Morris JM. First trimester screening of serum soluble fms-like tyrosine kinase-1 and placental growth factor predicting hypertensive disorders of pregnancy. Pregnancy hypertension. (2013) 3:215–21. doi: 10.1016/j.preghy.2013.04.119, PMID: 26103799

[24] Skrastad RB Hov GG Blaas HG Romundstad PR Salvesen KA. A prospective study of screening for hypertensive disorders of pregnancy at 11–13 weeks in a scandinavian population. . Acta Obstet Gynecol Scand. (2014) 93:1238–47. doi: 10.1111/aogs.12479, PMID: 25146367

[25] Li T Xu M Wang Y Wang Y Tang H Duan H. Prediction model of preeclampsia using machine learning based methods: A population based cohort study in China. Front Endocrinol (Lausanne). (2024) 15:1345573. doi: 10.3389/fendo.2024.1345573, PMID: 38919479 PMC11198873

[26] O'Gorman N Wright D Poon LC Rolnik DL Syngelaki A Wright A. Accuracy of competing-risks model in screening for pre-eclampsia by maternal factors and biomarkers at 11–13 weeks' Gestation. Ultrasound obstetrics gynecology: Off J Int Soc Ultrasound Obstetrics Gynecology. (2017) 49:751–5. doi: 10.1002/uog.17399, PMID: 28067011

[27] Ansbacher-Feldman Z Syngelaki A Meiri H Cirkin R Nicolaides KH Louzoun Y. Machine-learning-based prediction of pre-eclampsia using first-trimester maternal characteristics and biomarkers. Ultrasound obstetrics gynecology: Off J Int Soc Ultrasound Obstetrics Gynecology. (2022) 60:739–45. doi: 10.1002/uog.26105, PMID: 36454636

[28] Tiruneh SA Rolnik DL Selvaratnam R da Silva Costa F McLennan A Hyett J. External validation of the fetal medicine foundation model for preterm pre-eclampsia prediction at 11–14 weeks in an Australian population. Acta Obstet Gynecol Scand. (2025) 104:1774–82. doi: 10.1111/aogs.70002, PMID: 40600650 PMC12393981

[29] Zhao Q Li J Diao Z Zhang X Feng S Hou G. Early prediction of preeclampsia from clinical, multi-omics and laboratory data using random forest model. BMC Pregnancy Childbirth. (2025) 25:531. doi: 10.1186/s12884-025-07582-4, PMID: 40325391 PMC12051331

[30] Ekelund CK Rode L Tabor A Hyett J McLennan A. Placental growth factor and adverse obstetric outcomes in a mixed-risk cohort of women screened for preeclampsia in the first trimester of pregnancy. Fetal diagnosis Ther. (2021) 48:304–12. doi: 10.1159/000514201, PMID: 33789295

[31] O'Gorman N Wright D Syngelaki A Akolekar R Wright A Poon LC. Competing risks model in screening for preeclampsia by maternal factors and biomarkers at 11–13 weeks gestation. Am J obstetrics gynecology. (2016) 214:103.e1–.e12. doi: 10.1016/j.ajog.2015.08.034, PMID: 26297382

[32] Lee MS Cantonwine D Little SE McElrath TF Parry SI Lim KH. Angiogenic markers in pregnancies conceived through *in vitro* fertilization. Am J obstetrics gynecology. (2015) 213:212 e1–8. doi: 10.1016/j.ajog.2015.03.032, PMID: 25797229

[33] Darling AM McDonald CR Conroy AL Hayford KT Liles WC Wang M. Angiogenic and inflammatory biomarkers in midpregnancy and small-for-gestational-age outcomes in Tanzania. Am J obstetrics gynecology. (2014) 211:509 e1–8. doi: 10.1016/j.ajog.2014.05.032, PMID: 24881826 PMC4247823

