# Supplementary material for: Association of Placental Growth Factor with the risk of adverse pregnancy outcomes: a prospective cohort study in Chinese pregnant women
Source: Front Endocrinol (Lausanne). 2025 Oct 2;16:1674540. doi: 10.3389/fendo.2025.1674540 (PMC12527900; doi:10.3389/fendo.2025.1674540)
Supplement: Supplementary Material 1 — The diagnostic criteria and definitions of study outcome. [file DataSheet1.docx]

**Study outcome definition**

- **Preeclampsia:** defined as systolic blood pressure of 140 mmHg or more or diastolic blood pressure of 90 mmHg or more on two occasions at least 4 hours apart after 20 weeks of gestation in a woman with a previously normal blood pressure, with proteinuria of 300 mg or more per 24 hours urine collection or protein/creatinine ratio of 0.3 mg/dL or more or dipstick reading of 1+, or in the absence of proteinuria, new-onset hypertension with the new onset of any of the following: platelet count less than 100×10^9/L, serum creatinine concentrations greater than 1.1 mg/dL or a doubling of the serum creatinine concentration in the absence of other renal disease, elevated blood concentrations of liver transaminases to twice normal concentration, pulmonary edema, new-onset headache or visual symptoms unresponsive to medication and not accounted for by alternative diagnoses, fetal growth restriction.[1]
- **Gestational diabetes:** defined as a fasting plasma glucose level of 5.1 mmol/L or more, a 1-h level of 10.0 mmol/L or more or 2-h value of 8.5 mmol/L or more after 75-g oral glucose tolerance test.[2]
- **Gestational hypertension:** defined as systolic blood pressure of 140 mmHg or more or diastolic blood pressure of 90 mmHg or more. [1]
- **Ectopic pregnancy:** defined as a gestational sac that implants in a location that is not the uterus.[3]
- **Premature rupture of membranes:** the definition of PROM is rupture of membranes before the onset of labor.[4]
- **Placental abruption:** defined as premature separation of the placenta from the uterus, usually after 20 weeks gestation.[5]
- **Spontaneous abortion**: defined as pregnancy loss before 28 weeks gestation or with fetal weight below 1000g.[6]
- **Pacenta previa:** defined as implantation of the placenta over or near the internal cervical os after 28 weeks gestation.[7]
- **Large for gestational age**: infants whose weight is > the 90th percentile for gestational age.[10,11]
- **Small for gestational age:** infants whose weight is < the 10th percentile for gestational age.[10,11]
- **Preterm birth:** infants born before 37 weeks of gestation.[12]

**Reference:**

1. Pregnancy Hypertension Disorders Group of the Obstetrics and Gynecology Branch of the Chinese Medical Association. Guidelines for the Diagnosis and Treatment of Hypertensive Disorders during Pregnancy (2020) [J]. Chinese Journal of Obstetrics and Gynecology, 2020, 55(4): 227-238. DOI:10.3760/cma.j.cn112141-20200114-00039.
2. Obstetrics Group of the Obstetrics and Gynecology Branch of the Chinese Medical Association, Perinatal Medicine Branch of the Chinese Medical Association, Pregnancy with Diabetes Professional Committee of the China Maternal and Child Health Association. Guidelines for the Diagnosis and Treatment of Hyperglycemia during Pregnancy (2022) [Part One] [J]. Chinese Journal of Obstetrics and Gynecology, 2022, 57(1): 3-12. DOI:10.3760/cma.j.cn112141-20210917-00528.
3. American College of Obstetricians and Gynecologists' Committee on Practice Bulletins—Gynecology. ACOG Practice Bulletin No. 193: Tubal Ectopic Pregnancy. Obstet Gynecol. 2018 Mar;131(3):e91-e103. doi: 10.1097/AOG.0000000000002560. Erratum in: Obstet Gynecol. 2019 May;133(5):1059. PMID: 29470343.
4. American College of Obstetricians and Gynecologists’ Committee on Practice Bulletins—Obstetrics. Practice Bulletin No. 172: Premature Rupture of Membranes. Obstet Gynecol. 2016 Oct;128(4):e165-77. doi: 10.1097/AOG.0000000000001712. PMID: 27661655.
5. American College of Obstetricians and Gynecologists’ Committee on Practice Bulletins—Obstetrics. Practice Bulletin No. 172: Premature Rupture of Membranes. Obstet Gynecol. 2016 Oct;128(4):e165-77. doi: 10.1097/AOG.0000000000001712. PMID: 27661655.
6. Chinese Expert Consensus Group on the Diagnosis and Treatment of Spontaneous Miscarriage. Chinese Expert Consensus on the Diagnosis and Treatment of Spontaneous Miscarriage (2020 Edition) [J]. Chinese Journal of Practical Gynecology and Obstetrics, 2020, 36(11): 1082-1090. DOI:10.19538/j.fk2020110113.
7. Obstetrics Group of the Obstetrics and Gynecology Branch of the Chinese Medical Association. Guidelines for the Diagnosis and Treatment of Placenta Previa (2020) [J]. Chinese Journal of Obstetrics and Gynecology, 2020, 55(1): 3-8. DOI:10.3760/cma.j.issn.0529-567X.2020.01.002.
8. Endometriosis Collaborative Group of the Obstetrics and Gynecology Branch of the Chinese Medical Association. Guidelines for the Diagnosis and Treatment of Endometriosis [J]. Chinese Journal of Obstetrics and Gynecology, 2015(3): 161-169. DOI:10.3760/cma.j.issn.0529-567x.2015.03.001.
9. Organization WH. International statistical classification of diseases and related health problems, tenth revision. World Health Organization; 2004.
10. Lees CC, Stampalija T, Baschat A, da Silva Costa F, Ferrazzi E, Figueras F, Hecher K, Kingdom J, Poon LC, Salomon LJ, Unterscheider J. ISUOG Practice Guidelines: diagnosis and management of small-for-gestational-age fetus and fetal growth restriction. Ultrasound Obstet Gynecol. 2020 Aug;56(2):298-312. doi: 10.1002/uog.22134. PMID: 32738107.
11. Wang Ya, Wang Yuan, Tang Huirong, Zhang Yan, Dai Chenyan, Li Jie, Dai Yimin and Zheng Mingming. Establishment method and significance of birthweight curve and reference in single center (2023) [J]. Chinese Journal of Obstetrics and Gynecology, 2023, 58(5): 334-342. DOI:10.3760/cma.j.cn112141-20230120-00021.
12. Hu Yali. Guidelines for Clinical Diagnosis and Treatment of Preterm Birth (2014) [J]. Chinese Journal of Obstetrics and Gynecology, 2014(7): 481-485. DOI:10.3760/cma.j.issn.0529-567x.2014.07.001.
