# Supplementary material for: Association of Placental Growth Factor with the risk of adverse pregnancy outcomes: a prospective cohort study in Chinese pregnant women
Source: Front Endocrinol (Lausanne). 2025 Oct 2;16:1674540. doi: 10.3389/fendo.2025.1674540 (PMC12527900; doi:10.3389/fendo.2025.1674540)
Supplement: Supplementary file 2 [file Table1.docx]

**Table S1.** Sensitivity analysis of the effect of covariates on the association between PIGF level and maternal-fetal outcome

| **Outcome** | **Model 1** | | | **Model 2** | | | **Model 3** | | |
| --- | --- | --- | --- | --- | --- | --- | --- | --- | --- |
|  | **OR** | **95%CI** | ***P*** | **OR** | **95%CI** | ***P*** | **OR** | **95%CI** | ***P*** |
| **Normal PLGF Level** | | | | | | | | | |
| Gestational Diabetes | 1.00 | 0.99-1.00 | 0.895 | 1.00 | 0.99-1.01 | 0.902 | 1.00 | 0.99-1.01 | 0.885 |
| Gestational hypertension | 1.00 | 0.99-1.00 | 0.912 | 1.00 | 099-1.00 | 0.945 | 1.00 | 0.99-1.00 | 0.956 |
| Preeclampsia | 0.97 | 0.96-0.98 | **<0.001** | 0.97 | 0.96-0.98 | **<0.001** | 0.97 | 0.96-0.98 | **<0.001** |
| Preterm Preeclampsia (<37 w) | 0.96 | 0.94-0.98 | **<0.001** | 0.96 | 0.94-0.98 | **0.001** | 0.96 | 0.94-0.98 | **0.001** |
| Ectopic pregnancy | 1.00 | 0.99-1.01 | 0.847 | 1.00 | 0.99-1.01 | 0.838 | 1.00 | 0.99-1.01 | 0.841 |
| Placental Abruption | 0.99 | 0.98-1.01 | 0.486 | 0.99 | 0.98-1.01 | 0.487 | 0.99 | 0.98-1.01 | 0.496 |
| Premature rupture of membranes | 1.00 | 1.00-1.00 | 0.450 | 1.00 | 1.00-1.00 | 0.450 | 1.00 | 1.00-1.00 | 0.458 |
| Spontaneous abortion | 1.00 | 0.98-1.02 | 0.794 | 1.00 | 0.98-1.02 | 0.792 | 1.00 | 0.98-1.02 | 0.804 |
| Placenta praevia | 0.98 | 0.96-1.01 | 0.152 | 0.98 | 0.96-1.01 | 0.149 | 0.98 | 0.96-1.01 | 0.138 |
| Single live birth | 1.01 | 0.98-1.02 | 0.644 | 1.01 | 0.98-1.02 | 0.641 | 1.01 | 0.98-1.02 | 0.656 |
| Large for gestational age | 1.00 | 1.00-1.00 | 0.939 | 1.00 | 1.00-1.00 | 0.944 | 1.00 | 1.00-1.00 | 0.968 |
| Small for gestational age (< 10th) | 0.99 | 0.98-0.99 | **<0.001** | 0.99 | 0.98-0.99 | **<0.001** | 0.99 | 0.98-0.99 | **<0.001** |
| Small for gestational age (< 3th) | 0.98 | 0.97-0.99 | **<0.001** | 0.98 | 0.97-0.99 | **<0.001** | 0.98 | 0.97-0.99 | **<0.001** |
| Preterm birth (<37w) | 1.00 | 0.99-1.01 | 0.807 | 1.00 | 0.99-1.01 | 0.824 | 1.00 | 0.99-1.01 | 0.828 |
| **MoM value of PLGF** | | | | | | | | | |
| Gestational Diabetes | 1.06 | 0.92-1.22 | 0.448 | 1.06 | 0.92-1.22 | 0.455 | 1.06 | 0.92-1.22 | 0.432 |
| Gestational hypertension | 0.94 | 0.69-1.27 | 0.674 | 0.94 | 0.70-1.27 | 0.702 | 0.94 | 0.70-1.27 | 0.706 |
| Preeclampsia | 0.31 | 0.20-0.47 | **<0.001** | 0.31 | 0.20-0.47 | **<0.001** | 0.31 | 0.20-0.47 | **<0.001** |
| Preterm Preeclampsia (<37 w) | 0.23 | 0.10-0.56 | **0.001** | 0.23 | 0.10-0.57 | **0.001** | 0.23 | 0.10-0.57 | **0.001** |
| Ectopic pregnancy | 1.02 | 0.70-1.48 | 0.928 | 1.01 | 0.70-1.48 | 0.943 | 1.01 | 0.70-1.48 | 0.942 |
| Placental Abruption | 0.82 | 0.46-1.47 | 0.505 | 0.82 | 0.46-1.47 | 0.505 | 0.82 | 0.46-1.47 | 0.512 |
| Premature rupture of membranes | 0.95 | 0.85-1.07 | 0.405 | 0.95 | 0.85-1.07 | 0.404 | 0.95 | 0.85-1.07 | 0.412 |
| Spontaneous abortion | 0.92 | 0.48-1.76 | 0.806 | 0.92 | 0.48-1.76 | 0.807 | 0.94 | 0.49-1.77 | 0.836 |
| Placenta praevia | 0.62 | 0.28-1.38 | 0.244 | 0.62 | 0.28-1.38 | 0.243 | 0.61 | 0.27-1.37 | 0.229 |
| Single live birth | 1.18 | 0.58-2.38 | 0.652 | 1.18 | 0.58-2.38 | 0.652 | 1.16 | 0.58-2.31 | 0.681 |
| Large for gestational age | 1.07 | 0.95-1.21 | 0.243 | 1.07 | 0.95-1.21 | 0.244 | 1.08 | 0.96-1.21 | 0.212 |
| Small for gestational age (< 10th) | 0.67 | 0.54-0.83 | **<0.001** | 0.67 | 0.54-0.83 | **<0.001** | 0.66 | 0.53-0.82 | **<0.001** |
| Small for gestational age (< 3th) | 0.43 | 0.29-0.64 | **<0.001** | 0.43 | 0.29-0.64 | **<0.001** | 0.42 | 0.28-0.63 | **<0.001** |
| Preterm birth (<37w) | 1.01 | 0.82-1.25 | 0.915 | 1.02 | 0.82-1.25 | 0.886 | 1.02 | 0.82-1.26 | 0.874 |

*adjusted model1: adjusted for maternal age, BMI, mean arterial pressure, gestational week for PIGF testing, parity, smoking;

*adjusted model2: adjusted for covariates in model1 plus history of diabetes, history of hypertension;

*adjusted model3: adjusted for covariates in model2 plus history of renal disease, history of systemic lupus erythematosus (SLE)

Abbreviation: OR, odds ratio; CI, confidence interval; GW，gestational week.
