# Supplementary material for: Association of Placental Growth Factor with the risk of adverse pregnancy outcomes: a prospective cohort study in Chinese pregnant women
Source: Front Endocrinol (Lausanne). 2025 Oct 2;16:1674540. doi: 10.3389/fendo.2025.1674540 (PMC12527900; doi:10.3389/fendo.2025.1674540)
Supplement: Supplementary file 3 [file Table2.docx]

**Table S2** Interactions analysis of GDM on the association between PIGF level and adverse pregnancy outcomes

| **Outcome** | **GDM (Yes)** | | | **GDM (No)** | | |
| --- | --- | --- | --- | --- | --- | --- |
|  | **OR*** | **95%CI** | ***P*** | **OR*** | **95%CI** | ***P*** |
| **PIGF** | | | | | | |
| Preeclampsia | 0.98 | 0.96-1.01 | 0.165 | 0.97 | 0.96-0.98 | **<0.001** |
| Preterm Preeclampsia | 1.00 | 0.96-1.04 | 0.836 | 0.96 | 0.93-0.98 | **<0.001** |
| SGA<10th | 0.98 | 0.96-0.99 | **0.037** | 0.99 | 0.98-0.99 | **0.002** |
| SGA<3rd | 0.96 | 0.93-0.99 | **0.028** | 0.98 | 0.97-0.99 | **<0.001** |
| **MoM value of PIGF level** | | | | | | |
| Preeclampsia | 0.66 | 0.25-1.72 | 0.392 | 0.27 | 0.17-0.44 | **<0.001** |
| Preterm Preeclampsia | 1.01 | 0.36-1.20 | 0.985 | 0.15 | 0.05-0.42 | **<0.001** |
| SGA<10th | 0.44 | 0.21-0.92 | **0.030** | 0.69 | 0.55-0.86 | **0.001** |
| SGA<3rd | 0.24 | 0.06-0.86 | **0.029** | 0.46 | 0.30-0.69 | **<0.001** |

GDM, gestational diabetes mellitus; OR, odds ratio; CI, confidence interval. SGA<10th, birth weight below the 10th percentile for gestational age; SGA<3rd, birth weight below the 3rd percentile for gestational age. *adjusted model: adjusted for maternal age, BMI, mean arterial pressure, gestational week for PIGF testing;
