# Supplementary material for: Association of Placental Growth Factor with the risk of adverse pregnancy outcomes: a prospective cohort study in Chinese pregnant women
Source: Front Endocrinol (Lausanne). 2025 Oct 2;16:1674540. doi: 10.3389/fendo.2025.1674540 (PMC12527900; doi:10.3389/fendo.2025.1674540)
Supplement: Supplementary file 4 [file Table3.docx]

**Table S3** Summary of studies published in the past five years evaluating PIGF for risk stratification in pregnancy outcomes

| Source | Outcome | Population | Gestational window | Sample size | PIGF threshold | AUC | Sensitivity | Specificity | Pregnancies |
| --- | --- | --- | --- | --- | --- | --- | --- | --- | --- |
| Rachel A et al(1) | Preterm birth | Canada | 24-28 GW | 9037 | 290 pg/ml | 0.80 (0.75-0.85) | 0.65 (0.57-0.72) | 0.88 (0.87-0.88) | Single Pregnancies |
| Lihua Du et al(2) | Adverse pregnancy outcomes | China | 16-20 GW | 387 | 209 pg/ml | 0.87 (0.84-0.91) | 0.81 | 0.80 | Twin Pregnancies |
| Yimin Z et al(3) | Small for gestational age | China | 10-24 GW | 452 | 126.8 pg/ml | 0.83 | 0.75 | 0.80 | Single Pregnancies |
| Guijie Qi et al(4) | Preeclampsia | China | 24-36 GW | 141 | 215 pg/ml | 0.86 (0.80-0.93) | 0.86 (0.75-0.97) | 0.80 (0.72-0.88) | Twin Pregnancies |
| Shuai Li et al(5) | Discordant fetal growth | China | 24-28 GW | 860 | 187.5 pg/ml | 0.75 (0.65-0.85) | 0.77 | 0.71 | Monochorionic twin pregnancies |
| Shuai Li et al(5) | Discordant fetal growth | China | 24-28 GW | 860 | 252.5 pg/ml | 0.72 (0.66-0.78) | 0.65 | 0.69 | Dichorionic twin pregnancies |
| Alaa AI et al(6) | Ectopic pregnancy | Iraq | 5-10 GW | 240 | 15.5 pg/ml | 0.90 | 0.93 | 0.83 | Single Pregnancies |
| Parchem et al(7) | Composite neonatal outcome | USA | 20-41GW | 1112 | 100 pg/ml |  | 0.96(0.88-0.99) | 0.36(0.32-0.38) | Single Pregnancies |
| Kate E et al(8) | Preeclampsia | UK | 20-36 GW | 289 | 100 pg/ml | 0.79 (0.68-0.89) | 0.88 (0.68-0.97) | 0.59 (0.43-0.55) | Single Pregnancies |

Ref:

1. Gladstone RA, Ahmed S, Huszti E, McLaughlin K, Snelgrove JW, Taher J, et al. Midpregnancy Placental Growth Factor Screening and Early Preterm Birth. *JAMA Netw Open* (2024) 7(11):e2444454. Epub 2024/11/14. doi: 10.1001/jamanetworkopen.2024.44454.

2. Du L, Wang B, Zhang M, Bai J, Xu X, Wang N. Predictive Value of Placental Growth Factor Level for Adverse Pregnancy Outcome in Twin Pregnancies at Advanced Maternal Age. *Am J Transl Res* (2024) 16(11):6581-92. Epub 2024/12/16. doi: 10.62347/NFCD8953.

3. Zhang Y, Shao S, Xu Q, Qin J, Liu Z, Zhang X. The Correlation between Placental Growth Factor and Small for Gestational Age Infants: A Matched Case-Control Study. *J Matern Fetal Neonatal Med* (2024) 37(1):2428387. Epub 2024/11/18. doi: 10.1080/14767058.2024.2428387.

4. Qi G, Yao L, Liu Z, Guo W, Liu H, Zhang J, et al. Placental Growth Factor as a Predictive Marker of Preeclampsia in Twin Pregnancy. *J Perinat Med* (2025) 53(2):149-57. Epub 2024/12/20. doi: 10.1515/jpm-2024-0184.

5. Li S, Wu K, Zhou S, Yin B, Bai X, Zhu B. Predictive Value of Maternal Serum Placental Growth Factor Levels for Discordant Fetal Growth in Twins: A Retrospective Cohort Study. *BMC Pregnancy Childbirth* (2024) 24(1):10. Epub 2024/01/04. doi: 10.1186/s12884-023-06212-1.

6. Al Zubaidi A, Eid MM. The Diagnostic Utility of Placental Growth Factor in Ectopic Pregnancy. *Archives of gynecology and obstetrics* (2021) 304(3):833-8. Epub 2021/01/25. doi: 10.1007/s00404-021-05965-4.

7. Parchem JG, Brock CO, Chen HY, Kalluri R, Barton JR, Sibai BM, et al. Placental Growth Factor and the Risk of Adverse Neonatal and Maternal Outcomes. *Obstetrics and gynecology* (2020) 135(3):665-73. Epub 2020/02/07. doi: 10.1097/AOG.0000000000003694.

8. Duhig KE, Webster LM, Sharp A, Gill C, Seed PT, Shennan AH, et al. Diagnostic Accuracy of Repeat Placental Growth Factor Measurements in Women with Suspected Preeclampsia: A Case Series Study. *Acta Obstet Gynecol Scand* (2020) 99(8):994-1002. Epub 2020/02/06. doi: 10.1111/aogs.13818.
