# Supplementary material for: Association of Placental Growth Factor with the risk of adverse pregnancy outcomes: a prospective cohort study in Chinese pregnant women
Source: Front Endocrinol (Lausanne). 2025 Oct 2;16:1674540. doi: 10.3389/fendo.2025.1674540 (PMC12527900; doi:10.3389/fendo.2025.1674540)
Supplement: Supplementary file 5 [file Table4.docx]

**Table S4.** Logistic regression analysis on the association between PIGF level and maternal-fetal outcomes at different pregnancy periods

| **Outcome** | **18 -24 GW (N=1531)** | | | **28-34 GW (N=1449)** | | | **>35 GW (N=1154)** | | |
| --- | --- | --- | --- | --- | --- | --- | --- | --- | --- |
|  | **OR** | **95%CI** | ***P*** | **OR** | **95%CI** | ***P*** | **OR** | **95%CI** | ***P*** |
| **Normal PLGF Level** | | | | | | | | | |
| Gestational Diabetes | 1.00 | 0.99-1.01 | 0.581 | 1.00 | 0.99-1.01 | 0.728 | 1.00 | 0.99-1.01 | 0.770 |
| Gestational hypertension | 1.00 | 1.00-1.00 | 0.351 | 1.00 | 1.00-1.00 | 0.694 | 1.00 | 0.99-1.00 | 0.108 |
| Preeclampsia | 0.99 | 0.99-1.00 | **<0.001** | 0.99 | 0.99-1.00 | **<0.001** | 0.99 | 0.98-0.99 | **<0.001** |
| Preterm Preeclampsia (<37 w) | 0.99 | 0.99-1.00 | 0.006 | 0.99 | 0.99-1.00 | 0.004 | 0.89 | 0.65-1.20 | 0.442 |
| Ectopic pregnancy | 1.00 | 1.00-1.00 | 0.308 | 1.00 | 1.00-1.00 | 0.829 | 1.00 | 1.00-1.00 | 0.819 |
| Placental Abruption | 0.99 | 0.99-1.00 | 0.041 | 1.00 | 1.00-1.00 | 0.661 | 1.00 | 0.99-1.00 | 0.465 |
| Premature rupture of membranes | 1.00 | 1.00-1.00 | 0.830 | 1.00 | 1.00-1.00 | 0.987 | 1.00 | 1.00-1.00 | 0.682 |
| Placenta praevia | 1.00 | 1.00-1.00 | 0.428 | 1.00 | 1.00-1.00 | 0.222 | 0.99 | 0.97-1.00 | 0.093 |
| Endometriosis | 1.00 | 1.00-1.00 | 0.178 | 1.00 | 1.00-1.00 | 0.403 | 1.00 | 1.00-1.00 | 0.511 |
| Large for gestational age | 1.00 | 1.00-1.00 | **0.008** | 1.00 | 1.00-1.00 | **<0.001** | 1.00 | 1.00-1.00 | **0.041** |
| Small for gestational age (< 10th) | 1.00 | 1.00-1.00 | **<0.001** | 1.00 | 1.00-1.00 | **<0.001** | 1.00 | 1.00-1.00 | **0.005** |
| Small for gestational age (< 3th) | 1.00 | 0.99-1.00 | **<0.001** | 1.00 | 1.00-1.00 | **<0.001** | 1.00 | 0.99-1.00 | **0.003** |
| Preterm birth (<37w) | 1.00 | 1.00-1.00 | 0.086 | 1.00 | 1.00-1.00 | **0.036** | 0.98 | 0.97-1.00 | **0.018** |
| **MoM value of PLGF** | | | | | | | | | |
| Gestational Diabetes | 1.08 | 0.80-1.44 | 0.627 | 1.04 | 0.85-1.26 | 0.725 | 1.01 | 0.88-1.17 | 0.841 |
| Gestational hypertension | 1.30 | 0.76-2.24 | 0.336 | 0.92 | 0.59-1.43 | 0.701 | 0.65 | 0.37-1.14 | 0.134 |
| Preeclampsia | 0.27 | 0.14-0.55 | <0.001 | 0.11 | 0.04-0.27 | <0.001 | 0.18 | 0.06-0.49 | <0.001 |
| Preterm Preeclampsia (<37 w) | 0.22 | 0.07-0.74 | 0.014 | 0.05 | 0.01-0.39 | 0.004 |  |  |  |
| Ectopic pregnancy | 1.49 | 0.69-3.24 | 0.311 | 0.95 | 0.49-1.87 | 0.892 | 1.02 | 0.66-1.57 | 0.936 |
| Placental Abruption | 0.31 | 0.09-1.07 | 0.065 | 0.84 | 0.42-1.66 | 0.612 | 0.84 | 0.46-1.51 | 0.552 |
| Premature rupture of membranes | 1.06 | 0.85-1.31 | 0.624 | 1.03 | 0.90-1.19 | 0.626 | 1.05 | 0.95-1.15 | 0.349 |
| Placenta praevia | 1.44 | 0.72-2.91 | 0.306 | 0.51 | 0.16-1.59 | 0.243 | 0.07 | 0.00-1.41 | 0.083 |
| Endometriosis | 1.49 | 0.83-2.67 | 0.177 | 0.81 | 0.45-1.45 | 0.472 | 1.09 | 0.83-1.43 | 0.540 |
| Large for gestational age | 1.40 | 1.10-1.77 | **0.006** | 1.39 | 1.21-1.61 | **<0.001** | 1.09 | 0.98-1.21 | **0.1066** |
| Small for gestational age (< 10th) | 0.45 | 0.32-0.63 | **<0.001** | 0.45 | 0.33-0.60 | **<0.001** | 0.77 | 0.64-0.92 | **0.0048** |
| Small for gestational age (< 3th) | 0.26 | 0.14-0.48 | **<0.001** | 0.25 | 0.14-0.47 | **<0.001** | 0.43 | 0.26-0.71 | **0.0012** |
| Preterm birth (<37w) | 0.70 | 0.45-1.10 | **0.123** | 0.62 | 0.41-0.93 | **0.022** | 0.12 | 0.01-1.41 | 0.0925 |

*adjusted model: adjusted for maternal age, BMI, mean arterial pressure, gestational week for PIGF testing; OR, odds ratio; CI, confidence interval; GW，gestational week.
