# Supplementary material for: Association of Placental Growth Factor with the risk of adverse pregnancy outcomes: a prospective cohort study in Chinese pregnant women
Source: Front Endocrinol (Lausanne). 2025 Oct 2;16:1674540. doi: 10.3389/fendo.2025.1674540 (PMC12527900; doi:10.3389/fendo.2025.1674540)
Supplement: Supplementary file 6 [file Table5.docx]

**Table S5** Study population characteristics at different pregnancy stage

| **Characteristic** | **11-14 GW**  **(N=5310)** | **18-24 GW**  **(N=1531)** | **28-34 GW**  **(N=1449)** | **>35GW**  **（N=1154）** |
| --- | --- | --- | --- | --- |
| **Maternal Characteristic** | | | | |
| Maternal age, year* | 30.17±3.78 | 30.02±3.93 | 29.97±3.89 | 29.90±3.83 |
| Height, cm* | 161.95±4.78 | 161.87±4.76 | 161.85±4.75 | 161.93±4.75 |
| Pre-pregnancy weight, kg* | 57.47±8.81 | 57.00±8.55 | 56.99±8.68 | 56.99±8.69 |
| Body Mass Index, kg/m2* | 21.89±3.08 | 21.74±3.02 | 21.74±3.05 | 21.72±3.04 |
| Mean arterial pressure, mmHg* | 83.39±8.01 | 82.50±8.12 | 82.44±8.07 | 82.29±7.33 |
| Placental growth factor, pg/mL* | 32.38±44.52 | 35.84±28.05 | 35.73±28.60 | 35.85±29.49 |
| Gestational week at Delivery, week* | 38.91±2.17 | 38.97±1.83 | 39.09±1.41 | 39.44±1.02 |
| **Maternal Outcome** | | | | |
| Gestational Diabetes, n (%) | 515(9.70) | 145(9.78) | 136(9.59) | 109(9.57) |
| Gestational hypertension, n (%) | 175(3.30) | 30(2.02) | 31(2.19) | 23(2.02) |
| Preeclampsia, n (%) | 278(5.27) | 59(3.99) | 52(3.67) | 30(2.63) |
| Preterm Preeclampsia, n (%) | 64(1.22) | 21(1.42) | 16(1.13) | 1(0.09) |
| Ectopic pregnancy, n (%) | 77(1.45) | 12(0.81) | 12(0.85) | 10(0.88) |
| Placental Abruption, n (%) | 63(1.19) | 16(1.08) | 15(1.06) | 10(0.88) |
| Premature rupture of membranes, n(%) | 1116(21.02) | 297(20.04) | 292(20.59) | 207(18.17) |
| Spontaneous abortion, n (%) | 37(0.70) | 6(0.40) | 0(0.00) | 0(0.00) |
| Placenta praevia, n (%) | 48(0.90) | 16(1.08) | 10(0.71) | 5(0.44) |
| **Fetal outcome** | | | | |
| Endometriosis, n (%) | 100(1.88) | 21(1.42) | 20(1.41) | 15(1.32) |
| Single live birth, n (%) | 5268(99.23) | 1474(99.46) | 1417(99.93) | 1139(100.00) |
| NICU>24h, n (%) | 9(0.17) | 3(0.20) | 2(0.14) | 1(0.09) |
| Neonatal asphyxia, n (%) | 3(0.05) | 1(0.07) | 1(0.07) | 1(0.09) |
| Large for gestational age, n (%) | 681(12.89) | 191(12.93) | 182(12.83) | 141(12.38) |
| SGA<10th, n(%) | 655(12.43) | 181(12.25) | 170(11.99) | 129(11.33) |
| SGA<3rd, n (%) | 238(4.52) | 68(4.60) | 64(4.51) | 49(4.30) |
| Preterm birth (<37w), n (%) | 246(4.67) | 77(5.22) | 63(4.45) | 6(0.53) |

*Data are presented as mean±standard deviation. Spontaneous, Spontaneous pregnancy; OI, Ovulation Induction; IVF, In Vitro Fertilization; SGA<10th, birth weight below the 10th percentile for gestational age; SGA<3rd, birth weight below the 3rd percentile for gestational age.
