# Supplementary material for: Association of Placental Growth Factor with the risk of adverse pregnancy outcomes: a prospective cohort study in Chinese pregnant women
Source: Front Endocrinol (Lausanne). 2025 Oct 2;16:1674540. doi: 10.3389/fendo.2025.1674540 (PMC12527900; doi:10.3389/fendo.2025.1674540)
Supplement: Supplementary file 7 [file Table6.docx]

**Table S6** The predictive performance of PIGF for adverse offspring outcomes during 28-34 weeks of pregnancy (N=1449)

| **Outcome** | **PLGF** | | | **MOM of PLGF** | | |
| --- | --- | --- | --- | --- | --- | --- |
|  | **AUC (95%CI)** | **Se (95%CI)** | **Sp (95%CI)** | **AUC (95%CI)** | **Se (95%CI)** | **Sp (95%CI)** |
| Large for gestational age | 0.58 (0.56-0.61) | 0.38 (0.31- 0.46) | 0.75 (0.72 – 0.77) | 0.59 (0.57-0.62) | 0.37 (0.30 – 0.44) | 0.79 (0.76 – 0.81) |
| Preterm birth | 0.65 (0.62-0.67) | 0.43 (0.31- 0.56) | 0.84 (0.82 – 0.86) | 0.65 (0.62-0.67) | 0.39 (0.27 – 0.52) | 0.88 (0.86 – 0.90) |

AUC: Area Under the Curve; Se: Sensitivity; Sp: Specificity; PE: Preeclampsia; CI: Confidence Interval; GW: Gestational Weeks.
